# Data-driven discovery of Tsallis-like distribution using symbolic regression in high-energy physics

**DOI:** 10.1093/pnasnexus/pgae467

**Published:** 2024-10-17

**Authors:** Nour Makke, Sanjay Chawla

**Affiliations:** Qatar Computing Research Institute, HBKU, 34110 Doha, Qatar; Qatar Computing Research Institute, HBKU, 34110 Doha, Qatar

**Keywords:** model discovery, symbolic regression, Tsallis distribution, hadron production

## Abstract

The application of atificial intelligence (AI) in fundamental physics has faced limitations due to its inherently uninterpretable nature, which is less conducive to solving physical problems where natural phenomena are expressed in human-understandable language, i.e. mathematical equations. Fortunately, there exists a form of interpretable AI that aligns seamlessly with this requirement, namely, symbolic regression (SR), which learns mathematical equations directly from data. We introduce a groundbreaking application of SR on actual experimental data with an unknown underlying model, representing a significant departure from previous applications, which are primarily limited to simulated data. This application aims to evaluate the reliability of SR as a bona fide scientific discovery tool. SR is applied on transverse-momentum-dependent distributions of charged hadrons measured in high-energy-physics experiments. The outcome underscores the capability of SR to derive an analytical expression closely resembling the Tsallis distribution. The latter is a well-established and widely employed functional form for fitting measured distributions across a broad spectrum of hadron transverse momentum. This achievement is among the first instances where SR demonstrates its potential as a scientific discovery tool. It holds promise for advancing and refining SR methods, paving the way for future applications on experimental data.

**Significance Statement**

Symbolic regression (SR) has emerged as a primary form of interpretable AI for scientific applications because it is designed to learn mathematical equations directly from data. However, most existing studies on SR use synthetic data to demonstrate proof of concept. We provide the first conclusive result on the use of SR on hadron transverse-momentum distribution using real data. Remarkably, SR methods are able to infer a model similar to the Tsallis distribution, a well-known statistical distribution, from experimentally measured data in high-energy physics. The implications are profound as this opens the door for the discovery of deeper mathematical relationships hidden in large scientific data sets, including high-energy physics and beyond.

## Introduction

The hadronization mechanism remains poorly understood to fully describe hard-scattering processes that involve hadron production. It refers to the mechanism by which quarks and gluons form the hadrons that are observed in the final state. Hadronization is an intrinsically nonperturbative process, meaning that it is not calculable in perturbative Quantum Chromodynamics (QCD) theory, and its determination fully relies on experimental data. At present, it is only described in event generators by physically inspired phenomenological models with many free parameters tuned by comparison to experimental data. The most known are the Lund string model (1, 2) and the cluster model (3) respectively deployed in PYTHIA (4, 5) and HERWIG (6)^a^ physics event generators. Hadronization is an interesting application for machine learning (ML) whose aim is to learn models directly from data. Although we are not yet at this stage in physics, ML techniques have been newly introduced to hadronization studies to either replace one component in the existing models with neural networks (NN) or develop ML-based models (7–9). Such NN-based models are evidently trained using simulated datasets, and their performance is evaluated by comparing them to the established phenomenological models. On the other hand, the description of the measured hadron spectra requires the calculation of the cross sections of hadron production from scattered partons in perturbative QCD. Such calculations disregard detailed assumptions concerning hadron production mechanisms and utilize the universal fragmentation functions instead. Fragmentation functions (FFs) are interpreted as the probability of a particular parton transforming into a particular hadron, i→h (see, e.g. Ref. (10) and references therein). The current methodology relies on fitting experimental data with a given functional form at some fixed scale $s_{0}$ and then making predictions using the DGLAP evolution equation at other energies. The terms fragmentation and hadronization are often used interchangeably, but they refer to distinct mathematical tools used for different tasks.

The production of charged hadrons with large transverse momentum ($p_{T}$) (11–14) is crucial to unravel the nuclear structure of matter and the behavior of quarks and gluons at very high energies in hadronic collisions, as well as to determine the quark and gluon polarization in polarized proton–proton (*pp*) and lepton–proton (ℓp) collisions. Such measurements aim to potentially answer questions within QCD related to universality, factorization, etc. Measured hadron spectra are extensively used in global fits of fragmentation functions, commonly referred to as FFs parameterizations, to determine them. It is mandatory to question whether ML could assist in inferring models directly from data rather than fitting parameters of some predefined model structures and, most importantly, if the learned models could be expressed in physics language, i.e. the human language. Fragmentation was never tackled with ML techniques to the best of our knowledge, and this paper reports the first study that applies ML techniques, namely symbolic regression, to fragmentation using experimental data.

Symbolic regression, classified as interpretable ML by learning analytical models directly from data, is re-emerging as a powerful tool for scientific discovery. However, its application to experimental data is limited compared to other ML tools (15, 16), in particular to high-energy physics data and was deployed on synthetic datasets in the majority of SR applications. This paper reports the first application of ML on fragmentation by applying SR to experimentally measured distributions of charged hadrons as a function of hadron transverse momentum. It shows that a functional form similar to the Tsallis distribution could be learned directly from data.

**Fig. 1.**
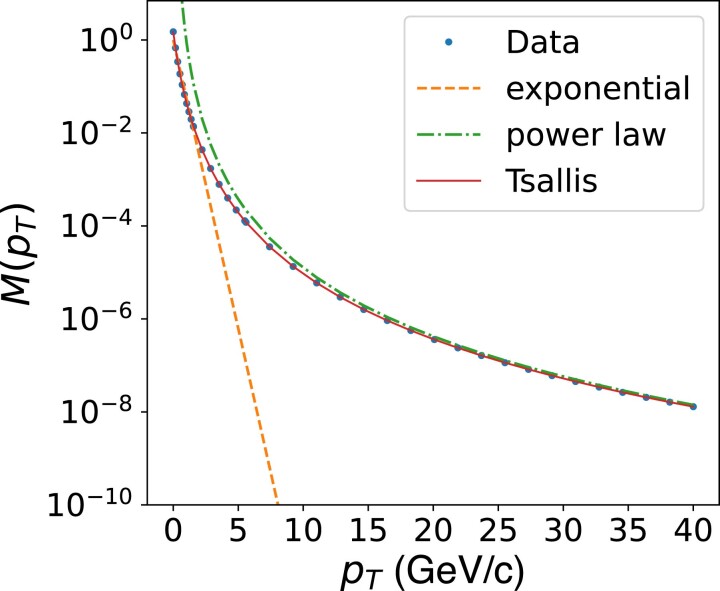
Sketch of a $p_{T}$-dependent distribution of charged hadrons showing a comparison between simulated data (markers) and (i) Boltzman–Gibbs statistical distribution (i.e. pure exponential function) $\text{exp}(-p_{T}/\alpha )$ (dashed line), (ii) a power-law function $p_{T}^{-n}$ (dash-dotted line), and (iii) the Tsallis distribution (Eq. 1) (solid line). $M(p_{T})$ denotes a measured physical observable versus $p_{T}$.

Transverse momentum distributions of charged hadrons are measured in different hard scattering processes, e.g. electron–positron annihilation, hadron collisions, and semi-inclusive deep-inelastic scattering, at significantly different center-of-mass energies, spanning increasing ranges in $p_{T}$ up to a few hundred GeV/c. The $p_{T}$-dependence of these distributions is essential because it reflects the interplay between the underlying hadron production mechanisms, as illustrated in the curves of Fig. 1. In the low-$p_{T}$ region, the distribution exhibits an exponential form $\text{exp}(-p_{T}/\alpha )$, highlighting that hadrons are predominantly produced through thermal processes following a statistical distribution described by the Boltzmann–Gibbs statistics. In the high-$p_{T}$ region, the distribution deviates from the exponential form, and exhibits instead a power-law behavior $p_{T}^{-n}$, typically associated with hard-scattering interactions, where *n* is often referred to as the “power-law” index. Whereas neither one of these functions fully captures the data across the entire range in $p_{T}$, the Tsallis distribution (17) provides an exceptionally accurate description. The latter was introduced by C. Tsallis, back in 1988, as a generalization (18) of the Boltzmann–Gibbs statistics and is represented by:


(1) $$F(p_{T})=A{\left[1-(1-q)\frac{\;p_{T}}{T}\right]}^{1/\left(1-q\right)}.$$

**Fig. 2.**
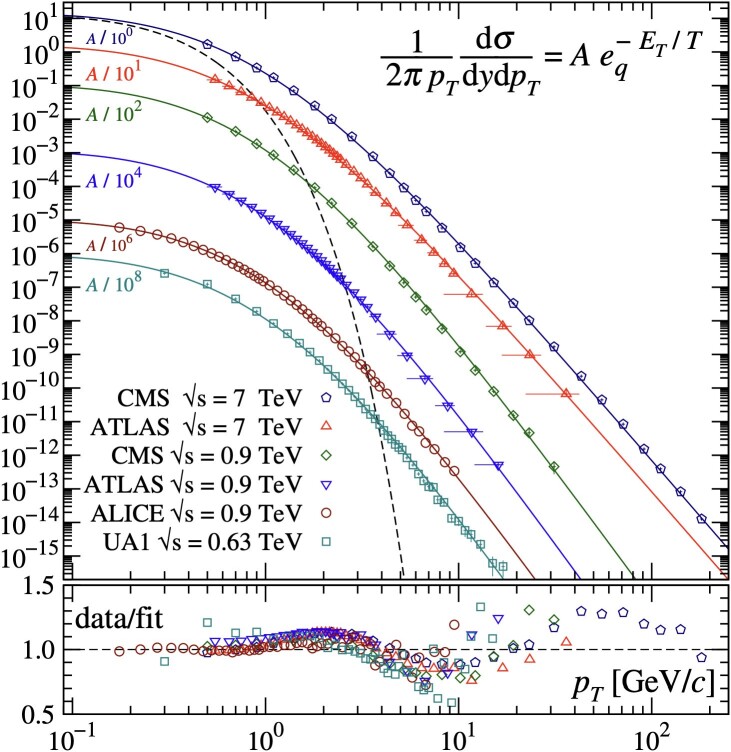
Comparison (22) of the Tsallis function (full line) and the experimental measurements of transverse momentum distributions of charged hadrons (markers) in *pp* collisions by different experiments. The dashed curve illustrates the corresponding Boltzmann–Gibbs distribution (i.e. purely exponential), which shows a significant discrepancy with experimental data. In contrast, the Tsallis adequately describes the data for the whole $p_{T}$ region at various center-of-mass energies. The data and the analytical curves have been divided by a constant factor for a better visualization as indicated. The data-to-fit ratios are shown at the bottom, and oscillating about unity.

Where *A* is a normalization constant, *T* could be physically interpreted as the temperature of a thermal distribution, and *q* is a real parameter. Making the identification q=1+1/n (or equivalently n=1/(1−q)) in this distribution (Eq. 1) is phenomenologically equivalent to the quasi-power law interpolating formula introduced by Hagedorn (19) and others (20, 21) for relativistic hard scattering:


(2) $$F(p_{T})=A{\left(1+\frac{\;p_{T}}{p_{0}}\right)}^{-n}$$

where $p_{0}=nT$. This approximates a purely exponential function $\text{exp}(-np_{T}/p_{0})$ at low $p_{T}$ and a pure power law function ($p_{T}^{-n}$) at large $p_{T}$. The extra parameter *q* in Eq. 1 takes the role of controlling the transition from exponential to power-law behavior. It provides an efficient parameterization of data, however experimental observations lead to questioning whether *q* can be considered a fundamental constant. The Tsallis function describes the shape of the measured $p_{T}$ spectra over the entire $p_{T}$ range. More interestingly, it was found to adequately describe over 14 decades of magnitude from the lowest to the highest $p_{T}$ spanned by the measured $p_{T}$-dependent hadron spectra in *pp* collisions at different energy scales, as shown in Fig. 2 from (22). Both Eqs. 1 and 2 have been widely used to fit $p_{T}$ spectra of charged hadrons measured in SPS, RHIC, and LHC experiments (23–31) and in phenomenological analyses of multiparticle production in high-energy processes, cf. (32–43) and references therein, where the numerical values of the free parameters (A,T,q/n) in Eqs. 1 and 2 are determined from fits to datasets.

## SR method and dataset

### Symbolic regression method

**Fig. 3.**
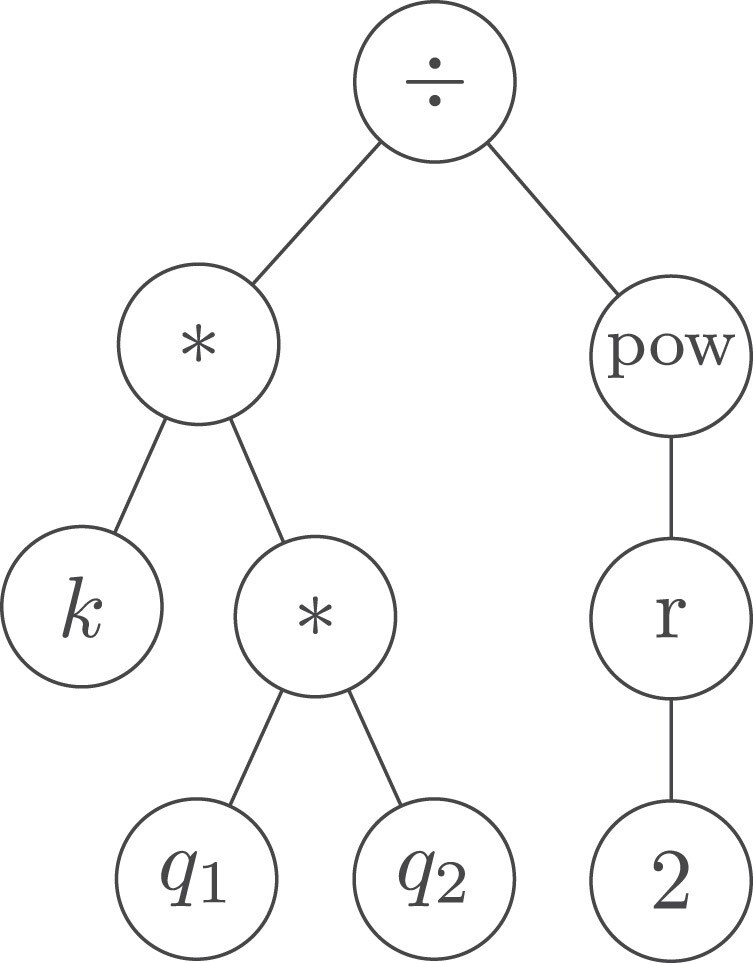
An exemplary expression-tree structure of the Coulomb force $F_{e}=kq_{1}q_{2}/r^{2}$, which measures the interaction between two electrically charged particles $q_{1}$ and $q_{2}$ distant by *r*. The numeral “2” refers to the exponent of the power operator.

In symbolic regression, both models’ structure and parameters are simultaneously learned. SR reduces to discovering a unary-binary tree (44) of mathematical symbols compatible with data. In such trees, internal nodes represent mathematical functions, and leaf nodes represent variables or constants, as illustrated in Fig. 3. This representation is important because any tree can be traversed into a unique sequence of symbols in prefix notation, referred to as the Polish notation (45). For example, the equation f(x)=0.5*exp(x) can be expressed as {*,0.5,exp,x}. This allows the employment of sequence-to-sequence ML-based models in the framework of SR. The optimization problem in SR is defined over the space of mathematical expressions, composed from a user-defined set of allowable mathematical operators, commonly referred to as a “library,” e.g. L={add,sub,mul,etc.}. The SR problem is nontrivial given the discrete nature of the search space, and, in general, it has been shown to be an “NP-hard” problem (46). To further discuss this point, consider the Coulomb force formula, $F_{e}=kq_{1}q_{2}/r^{2}$, consisting of nine symbols, and a library including 20 mathematical operations, e.g.


$$\begin{array}{rl} L & =\{+,-,*,÷,\cos,\sin,\tan,\text{exp},\log,\mathrm{sqrt},\mathrm{inv},\\ & \;\mathrm{Abs},\mathrm{pow}2,\mathrm{pow}3,\mathrm{pow}4,x,y,c,1,2\} \end{array}$$

Fitting the data set with a naive brute-force search will have to consider up to $20^{9}=51.2\times 10^{10}$ candidate solutions without accounting for the optimization of the numerical constant *c*, which is identified as Coulomb’s constant *k*. It is worthy of note that any equation can generally be expressed in infinite ways^b^ and thus could be more or less complex; however, ML-based models are trained to learn succinct mathematical equations through training datasets.^c^ The number of trials thus increases with model complexity (i.e. length of formula). SR can be tackled with various approaches, including genetic algorithms and deep learning, among others, as reviewed in (47, 48).

We specifically choose the NeSymReS (49) SR method based on an encoder–decoder transformer architecture, the latter was introduced by Vaswani *et al.* (50) in NLP^d^ to learn the context in text data by introducing attention blocks into NNs’ architecture. The outstanding performance of transformers has quickly expanded their use beyond NLP to sequential data, including time-series data. In the context of SR, transformers are configured as set-to-sequence models, i.e. they input a set of numerical data points and output a sequence of mathematical symbols. This results in a crucial difference in NeSymReS represented by the fact that the encoder itself is based on a set transformer to ensure permutation invariance, i.e. the ordering of the dataset does not impact the target sequence. We explain NeSymReS through an example. Consider the equation y=3*sin(x)+2*x with *n* data points. The (numerical) dataset is converted into a 16-bit binary representation and then passed to the encoder as a set of (2,16) matrices consisting of *n* elements, where “2” denotes the number of variables, i.e. *x* and *y*. The Encoder applies a set transformer and multi-head pooling operations to output a high-dimensional latent embedding vector (*z*) of the set, and the actual size is determined by hyperparameters tuning to achieve best performance during training. The ground-truth equation is converted into its skeleton form, i.e. e=∘*sin(x)+∘*x, and represented in a sequential prefix form^e^ with positional encoding following the standard decoder architecture introduced in (50). The decoder is then fed the skeleton with the positional embedding and the latent representation (e,z), and outputs a probability distribution over all the valid tokens, $P(e_{k+1}\;|\;e_{1\;:\;k},z)$, where (k+1) denotes the token to be predicted and (1:k) denotes the previously predicted tokens. The loss function is the standard cross-entropy loss. Its value is backpropagated through both the decoder and the encoder blocks, and their weights are updated. This process repeats across all training examples. The model is trained to reduce the average loss between the skeletons of the predicted equation and the ground-truth one. For pretraining, equation skeletons (with constant placeholders placed randomly inside the expressions) and the inputs are generated from a sampling distribution $P_{e,X}$, where $e\equiv f_{e}\;:\;R^{d_{x}}\to R^{d_{y}}$ and $X\equiv \{x_{i}{\}}_{i=1}^{n}$. The input’s size is varying, and data points are not required to be *i.i.d*, neither within *X* nor across examples or batches (49). Notably, the numerical constants and number of points per equation are continuously sampled at every training iteration, making it impossible to see exactly any of the test data at training time. During inference, a new data set (X,y) is encoded into *z*, which is then passed through the decoder to create a sequence of symbols in an auto-regressive manner, i.e. each symbol generated is then appended to the input, and the next symbol is generated based on the new context. Finally, the generated skeleton equation is converted into a “proper” equation by replacing the constant tokens (“°”) with their numeric counterparts using nonlinear optimization whose objective function is the root mean square error.

The choice of a transformer-based SR is mainly driven by the fact that learning the context in data holds significant meaning in physics, particularly in light of the causal nature of physical phenomena, where capturing correlations among variables is crucial. Two pretrained NeSymReS models are available. They can be directly used for inference without requiring the model to be trained from scratch for each new problem. This study uses the model pretrained on 100 million datasets (X,y,e). Its parameters are loaded into the model, which is then called for in every inference problem. The original library of operators in NeSymReS is deployed without constraints. There could be some constraints in NeSymReS’s library. However, a possible bias has been limited because the library includes all basic arithmetic operations and mathematical functions such that functions composed of basic operations can be constructed from the library. For example, the sigmoid function $1/(1+e^{-x})$ is not part of it; however, all its components are included, i.e. {+,÷,exp,x,1}. Finally, as previously mentioned, the numerical constants and number of points per equation are continuously sampled at every training iteration, making it impossible to see exactly any of the test data at training time, which reduces a possible bias due to the use of a pretrained transformer.

### Experimental dataset

The primary dataset used in this application is the semi-inclusive measurement of deep-inelastic scattering (SIDIS, $\ell p\to \ell^{\prime }hX$), where a lepton (ℓ) scatters off a target proton (*p*) and exchanges a virtual photon, a final-state hadron (*h*) is detected in coincidence with the scattered lepton ($\ell^{\prime }$), and *X* represents any other hadrons produced in the scattering. Experimental observables are the differential multiplicities of charged hadrons ($M^{h}$), defined by the ratio of inclusive ($\ell p\to \ell^{\prime }X$) and semi-inclusive ($\ell p\to \ell^{\prime }hX$) DIS cross sections. They are measured as a function of the square of the hadron’s transverse momentum $p_{T}^{2}$, defined as the transverse projection of hadron’s momentum vector with respect to the virtual photon direction, across simultaneous intervals of three kinematic variables: the Bjorken scaling variable *x*, the squared of the four-momentum of the exchanged virtual photon $Q^{2}$, and the hadron fractional energy *z*. The full dataset consists of 81 kinematic intervals, resulting in a total of 4918 experimental data points as reported in (31). The observed patterns reveal that the shape of the hadron multiplicities exhibits substantial sensitivity to variations in *x*, while its dependence on $Q^{2}$ is comparatively weaker. In light of these observations, various dataset configurations are considered by selecting distinct sets of variables. The selection of this specific dataset is underpinned by both physical and technical considerations. From a physics standpoint, the hadron fragmentation studied through the measurement of $p_{T}$ distributions is poorly understood and lacks a comprehensive theoretical framework. Moreover, the conventional approach to fitting these data employs a functional form that is highly efficient, relatively simple, and applicable across various energy scales. On the technical front, two key considerations come into play. The dataset’s richness is notable, encompassing multiple subsets that reveal a consistent fundamental structure while spanning diverse regions in the phase space. This mirrors multiple instances of SR to the same problem but with distinct data points. Secondly, the effectiveness of the results can be easily verified for generalization within the same dataset (i.e. across different intervals) and extended to other datasets within the same category (i.e. $p_{T}$-dependent distributions of hadrons measured in other processes/experiments). This approach is facilitated by the expectation that hadron fragmentation exhibits universality. In addition to the SIDIS dataset, we consider $p_{T}$ spectra of charged hadrons measured at the ALICE experiment at the Large Hadron Collider at CERN (11–13) at different center-of-mass energies, $\sqrt{s}=0.9,2.76$, and 7 TeV.

Successful results are only reported since the primary purpose of this paper is to investigate the credibility of SR as a scientific discovery tool by applying it to experimental (or observed) data rather than comparing the performance of existing SR methods. The reported results are obtained using the NeSymReS (49) method in inference mode.

## Experimental setup

### Data partition

The transverse-momentum-dependent charged hadrons multiplicities in SIDIS provide access to the quarks’ intrinsic transverse momenta and their dependence upon *x*, $Q^{2}$, and *z*; they are thus measured in intervals of these kinematic variables. To align the analysis with these goals, the full dataset $D\equiv \{x,Q^{2},z,p_{T}^{2},M^{h}\}$ is divided into subsets^f^; each one corresponds to $M^{h}(p_{T}^{2};I_{i})$ in an individual interval $I_{i}=(x,Q^{2},z{)}_{i}$ of the phase space. This allows to check the consistency of SR performance across different regions of the kinematic phase space.

### Training and test data

**Fig. 4.**
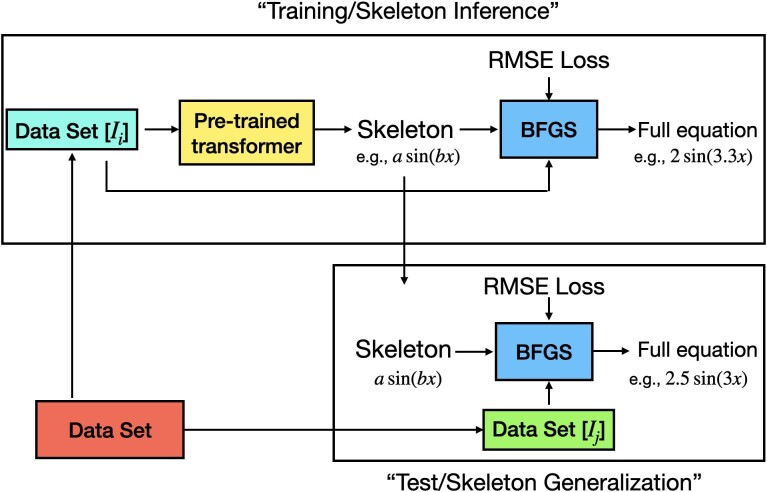
“Training” and “test” phases as defined in the present study that applies the pretrained transformer model NeSymReS (49) on experimental physics data (31). Training is described as a skeleton inference phase and “test” as a skeleton generalization phase. During training, a data subset $M^{h}(p_{T}^{2};I_{i})$ measured in a given kinematic interval $I_{i}=(x,Q^{2},z{)}_{i},\;i\in \{i_{1},\ldots ,i_{n}\}$ is input to the pretrained model, and infers an equation skeleton $f_{e}(p_{T}^{2},I_{i})$. The values of the numerical constants in the skeleton are determined in the subsequent optimization step using BFGS to learn the full equation. The generalizability of the inferred skeleton is checked in other intervals of the kinematic phase space using test data, $I_{k}=(x,Q^{2},z{)}_{k},\;i\in \{k_{1},\ldots ,k_{m}\}$. In the test phase, the same equation skeleton $f_{e}(p_{T}^{2},I_{i})$ is used to learn its parameters.

The standard ML training procedure, in which a model’s weights are updated to minimize the empirical risk, does not apply in the present study, given that a pretrained transformer network is used. Therefore, the terms “training” and “test” data are specifically defined for the purpose of this study, as explained in the illustration of Fig. 4. “Training” is regarded as a skeleton inference phase, and “test” as a skeleton generalization phase. The validation procedure for training comprises two steps: (i) An equation skeleton $f_{e}(p_{T}^{2};I_{i})$ is inferred from a training data subset $M^{h}(p_{T}^{2};I_{i})$ using the pretrained transformer model, and (ii) numerical values of the skeleton’s constants are then determined to learn the full equation in the optimization step using BFGS. This procedure is applied to data in all kinematic intervals. Only intervals $I_{i}=(x,Q^{2},z),\;i\in \{i_{1}\ldots i_{n}\}$ where SR infers an equation that fairly describes data are selected as “training” data. The test phase focuses on checking the generalizability of the learned skeleton. Therefore, the same skeleton $f_{e}(p_{T}^{2};I_{i})$ is used to learn the full equation by optimizating the values of the numerical constants against the measured data for all remaining kinematic intervals $I_{k}=(x,Q^{2},z),k\in \{k_{1},\ldots ,k_{m}\}$. Test data thus refers to these intervals $I_{k},k\in \{k_{1},\ldots ,k_{m}\}$.

## Results

We consider different ranges in $p_{T}^{2}$, (i) the entire $p_{T}^{2}$-range (Section ‘Full $p_{T}$ range’) and (ii) a truncated $p_{T}^{2}$-range (Section ‘Truncated $p_{T}$ range’) to check the consistency of the learned models with the observed dependencies upon $p_{T}$. The SR input $d_{i}\in D$ is defined in each section, and the functional forms that are obtained by independently applying NeSymReS to these subsets $d_{i}$ are reported on the basis of the most frequently learned functions, and they are presented in each subsection. In the following, $p_{T}^{2}$ is represented by “*u*” for an easier reading.

### Full $p_{T}$ range

#### 1D configuration

**Fig. 5.**
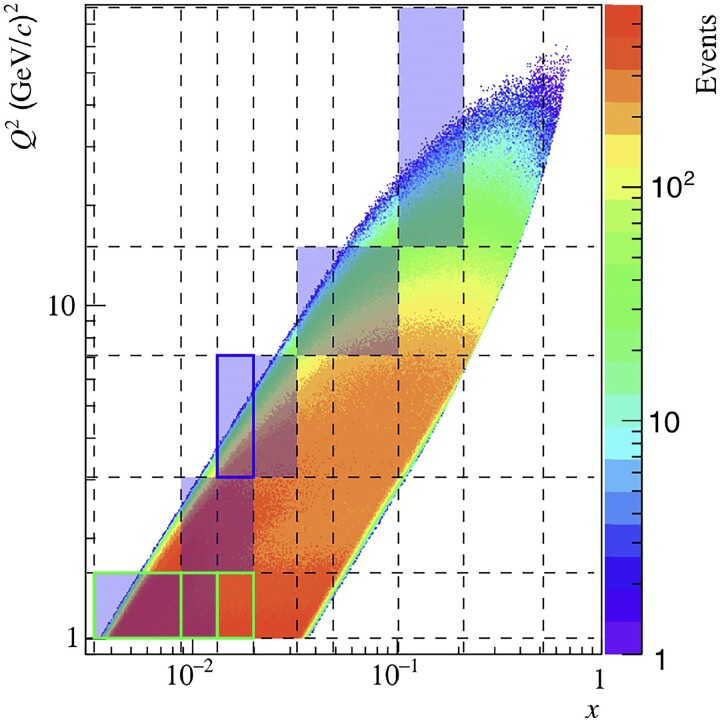
Kinematic distribution of data (scattering events) from the original analysis reported in (31) in ($x,Q^{2}$) intervals. The dashed lines delimit the (*x*, $Q^{2}$) intervals. The drawn boxes represent ($x,Q^{2}$) intervals where the SR model (cf. Eq. 6) is learned, for the full $p_{T}^{2}$ range, in the second *z* interval (0.3<z<0.4, shaded boxes), the third *z* interval (0.4<z<0.6, ($x_{3},Q_{3}^{2}$)), and the fourth *z* interval (0.6<z<0.8, ($x_{1},x_{2},x_{3};Q_{1}^{2}$)). These highlighted intervals represent the training data sets. All other (*x*, $Q^{2}$) intervals are, hence, test data sets, which are used to test the validity and generalizability of the learned SR model through a fit.

The full dataset comprises 81 separate subsets, i.e. $d_{i}\equiv \{p_{T}^{2},M^{h}\}$, which are independently passed to the SR algorithm. Table 1 summarizes the most frequently learned functions along with the ranges in the loss values. Although the learned functions do not provide a reasonable description of the data in most intervals, they commonly share a basic structure that may be written as:


(3) $$f(u)\propto {\left(1+cu^{n}\right)}^{-1},\;n=2,3,4$$

where *c* denotes a numerical constant and *n* is a power index. This functional form is obtained in 57 out of 81 intervals, covering different regions of the kinematic space. Table 2 reports on the results in a more detailed manner, where the most frequently learned functions are presented in each of the four intervals of *z*. Notably, the complexity (i.e. the length of a formulae’s sequence) among all learned functions is very similar, and the learned function associated with the lowest values of the loss function is found to be $f_{3}(u)=c_{0}/(1+c_{1}u^{3})$. It is noteworthy that $f_{7,8}$ are reported in Table 2 for completeness, and they are not considered because they do not fulfill dimensional analysis requirements. The loss values are not shown in Table 2 for simpler reading; however, the lowest values are reported in its caption. Figure 5 illustrates the kinematic phase space of the original dataset used in the analysis of (31). It highlights the $\left(x,Q^{2}\right)$ intervals where $f_{3}(u)$ was learned using SR in the intervals $z_{2}$ (shaded blue boxes), $z_{3}$ (blue boxes), and $z_{4}$ (green boxes). The multiplicities $M^{h}(p_{T}^{2})$ in these intervals represent the “training” data, whereas they represent the “test” data in the remaining $\left(x,Q^{2}\right)$ intervals. The latter allows for checking the generalizability of the learned model to “unseen data,” i.e. data that are not part of the training, by fitting the corresponding multiplicities using $f_{3}(u)$ where $c_{0}$ and $c_{1}$ are considered free fit parameters.

**Table 1.** Results of mathematical expressions learned by SR (NeSymReS (49)) on physics data (31) where the full $p_{T}^{2}$ range is considered.

Name	Expression	Complexity	Loss range	NOF
$f_{1}(u)$	$1/(1+cu^{3})$	9	[1.17–3]	17
$f_{2}(u)$	$1/(1+cu^{2})$	9	[1.9–4.35]	9
$f_{3}(u)$	$c_{0}/(1+c_{1}u^{3})$	9	[0.018–0.025]	13

Complexity represents the length of expressions’ sequences, the loss range shows the minimum and the maximum values of the loss values obtained for the shown expressions, and NOF denotes the number of times $f_{i}$ is learned. ($u\equiv p_{T}^{2}$).

**Table 2.** Results of mathematical expressions learned by SR (NeSymReS (49)) on SIDIS data (81 subsets) physics data (31), presented in different intervals of the variable *z* (first row).

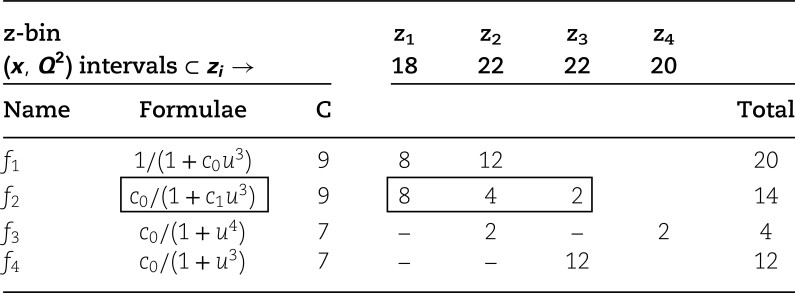

The second row presents the number of $\left(x,Q^{2}\right)$ intervals in each interval of *z*. “C” refers to the complexity of an expression defined by its sequence’s length. The full $p_{T}^{2}$ range is considered in the input data. The numbers in the box refer to the learned functions associated with the lowest loss values, which are found to be in the range [0.018,0.27] for $z_{2}$, $2\times 10^{-3}$ for $z_{3}$ and $[4,9]\times 10^{-4}$ for $z_{4}$. ($u\equiv p_{T}^{2}$).

**Fig. 6.**
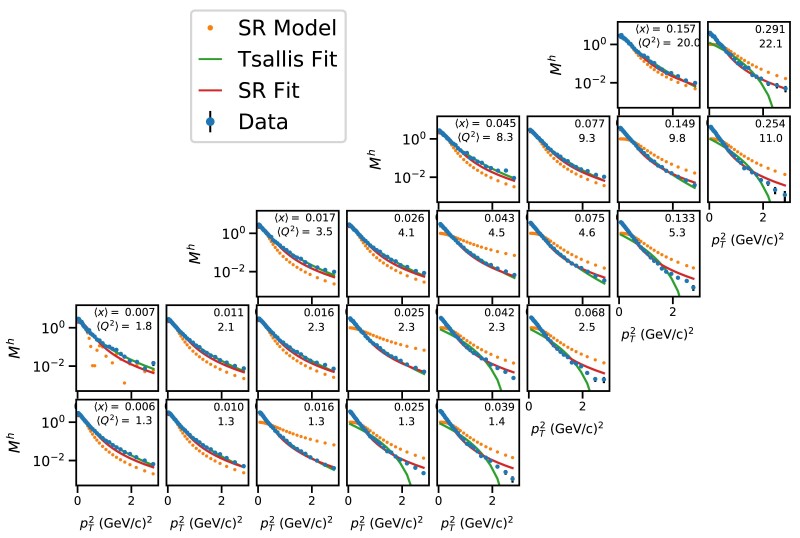
Differential multiplicities (31) of charged hadrons as a function of $p_{T}^{2}$ in ($x,Q^{2}$) intervals with z∈[0.3,0.4. Data are compared to learned SR models and to data fits using the Tsallis model, cf. Eq. 1 and the top SR model, cf. $f_{3}(u)=c_{0}/(1+c_{1}u^{3})$ in Table 1. Statistical uncertainties are considered in the fits.

Figure 6 presents the multiplicities of charged hadrons as a function of $p_{T}^{2}$, in ($x,Q^{2}$) intervals in the second interval of *z*, in comparison with the SR models that are independently learned from individual subsets ($d_{i}$, orange markers). Two fits are performed on individual data subsets using the Tsallis model (Eq. 1) and the SR model $f_{3}(u)=c_{0}/(1+c_{1}u^{3})$ reported in Tables 1, 2. They are shown in comparison to data. Whereas $f_{3}(u)$ is learned in few among all ($x,Q^{2}$) intervals, it generalizes well across all ($x,Q^{2}$) intervals. Both Tsallis and SR models provide a fair description of the data; the SR model better describes data in the range of large *x* at fixed values of $Q^{2}$, despite that it has only two free parameters with respect to Tsallis. It is noteworthy that the worst case is the SR model learned in the interval ⟨x⟩=0.007 and $\langle Q^{2}\rangle =1.8$ (cf., first column, second row), where the learned function is $f(u)=c_{0}\;*\;\sin\;(c_{1}*u{)}^{2}/u^{2}$. This represents one of some cases where the models learned by SR are not meaningful, and thus, they are not reported. In addition, the learned SR models in the lowest ranges of ($x,Q^{2}$) better describe data than those learned in the highest ranges of ($x,Q^{2}$). They respectively correspond to $f_{2}$ and $f_{1}$, cf. Table 1.

Another fit to experimental data is performed using the best-learned function ($f_{3}$ in Table 1) while treating the power index of *u* as a free fit parameter, i.e.:


(4) $$f(u)=c_{0}(1+c_{1}\;*\;u^{c_{2}}).$$

This resulted in $c_{2}>2.5$ in nearly half of the total number of $\left(x,Q^{2},z\right)$ intervals and an average value of 2.55 over all intervals. This result supports the finding that $f_{3}(u)=c_{0}/(1+u^{3})$ is generalizable to all intervals, e.g. where $f(u)\propto 1/(1+u^{2})$ was learned, as illustrated in the fits in Fig. 6.

#### 2D configuration

$D\equiv \{Q^{2},p_{T}^{2},M^{h}\}$ , resulting in a total of 32 separate data subsets (8 *x* intervals, 4 *z* intervals). The top performing functions, which provide a fair description of data, are two:


(5) $$\begin{array}{rl} \;f_{1}(u,Q^{2}) & =(1+cu^{3}{)}^{-1}\\ f_{2}(u,Q^{2}) & =\frac{Q^{2}}{\left(u(Q^{2}+c_{0}u^{2})\right)}=u^{-1}{\left(1+\frac{c_{0}u^{2}}{Q^{2}}\right)}^{-1} \end{array}$$

The function $f_{1}$ (same $f_{1}$ in Table 1) is independent of the additional variable $Q^{2}$, whereas $f_{2}$ exhibits a slight dependence on $Q^{2}$. $f_{2}$ is learned in only 5 (z,x) intervals out of 32 but is the best in terms of data description. In addition, it generalizes well to other intervals and outperforms the models individually learned by SR. Finally two “2D-configurations” are considered: $\left(Q^{2},p_{T}^{2}\right)$ and $\left(x,p_{T}^{2}\right)$. Whereas SR models obtained using the $\left(Q^{2},p_{T}^{2}\right)$ configuration provide good results, models learned in the $\left(x,p_{T}^{2}\right)$ configuration are inconclusive. This can be interpreted by the observation that the slope of the $p_{T}^{2}$-dependence of the distributions changes with increasing *x*, whereas its dependence on $Q^{2}$ is rather weak. Therefore, merging data from different $Q^{2}$ intervals improves the results on SR models.

### Truncated $p_{T}$ range

The entire $p_{T}^{2}$ range covered in the measured hadron multiplicities is decomposed into low-$p_{T}^{2}$ and high-$p_{T}^{2}$ ranges, resulting in three ranges. SR (NeSymReS) is independently applied on each subset within D with a truncated range in $p_{T}^{2}$, and results are reported in the following:

#### $p_{T}^{2}<0.5\;(\mathrm{GeV}/c{)}^{2}$

The best-learned expressions are exponential functions, e.g. $\text{exp}(-u)/u^{2}$, $c_{0}\text{exp}(c_{1}u)/u$, exp(−cu)/u. Notably, an exponential form is not frequently learned, although expected, and a combination of exponential and trigonometric functions, e.g. exp(−sin(cu))/u, $\text{exp}(-\tan\;(cu+c_{1}))/u$, is rather learned in numerous cases with a fair data-fit match. Nevertheless, many of these functions are deemed inconsistent from a dimensional analysis perspective, leading to their exclusion, also because of the lack of relevance of trigonometric functions in the context of the studied problem. The trigonometric functions were explicitly retained in the library to add complexity to the SR problem and to investigate the credibility of SR in the worst-case scenario, where the search space is maximized and all function types are permitted.

#### $p_{T}^{2}>0.5\;(\mathrm{GeV}/c{)}^{2}$

The top-performing expressions obtained using SR by discarding the low-$p_{T}^{2}$ region (i.e. $p_{T}^{2}<0.5\;(\mathrm{GeV}/c{)}^{2}$) are summarized in Table 3 along with the ranges in the loss values obtained for each $f_{i}$. The function that best describes data across various *z* ranges, and for which the loss values are the lowest, is ($f_{3}$ in Table 3):


(6) $$f(u)=c_{0}(1+c_{1}u^{3}{)}^{-1}.$$

**Table 3.** Results of mathematical expressions learned by SR (NeSymReS (49)) for physics data (31) with a truncated $p_{T}^{2}$ range ($p_{T}^{2}>0.5\;(\mathrm{GeV}/c{)}^{2}$). ($u\equiv p_{T}^{2}$).

Name	Expression	Loss range	NOF
$f_{1}(u)$	$1/(1+c_{0}u^{3})$	[0.02,3]$\;\times \;10^{-3}$	20
$f_{2}(u)$	$c_{0}/(1+u^{3})$	[0.3,1.5]$\;\times \;10^{-3}$	12
$f_{3}(u)$	$c_{0}/(1+c_{1}u^{3})$	[2,30]$\;\times \;10^{-5}$	14

For which the values of the loss function are significantly better up to 2 orders of magnitude difference) than those obtained for $f_{1}(u)\propto (1+c_{0}u^{3}{)}^{-1}$. This function was also learned by considering the full $p_{T}^{2}$ range ($f_{3}$ in Table 1), except that it describes significantly better data with $p_{T}^{2}>0.5\;(\mathrm{GeV}/c{)}^{2}$. This observation is interesting; NeSymReS could correctly learn the basic structure of the underlying model regardless of the data-fit match quality, as can be concluded from the comparison of Tables 1 and 3. Tables 4 and 5 present the functions learned by SR using a truncated $p_{T}^{2}$ range in details. In Table 4, the most frequently learned functions are presented for each interval of *z* along with the loss values. Table 5 summarizes the results obtained in each interval in *z*.

**Table 4.** Results of mathematical expressions learned by SR (NeSymReS (49)) on physics data (31), presented in different intervals of the variable *z* along with the loss values.

Interval	Expression	Loss range	NOF
$z_{1}$	$1/(1+c_{0}u^{3})$	[8,30]$\;\times \;10^{-4}$	8
	$c_{0}/(1+c_{1}u^{3})$	[3,30]$\;\times \;10^{-5}$	8
$z_{2}$	$1/(1+c_{0}u^{3})$	[0.2,6.8]$\;\times \;10^{-4}$	12
	$c_{0}/(1+c_{1}u^{3})$	[5,24]$\;\times \;10^{-5}$	4
$z_{3}$	$c_{0}/(1+c_{1}u^{3})$	[2-8]$\;\times \;10^{-5}$	2
	$c/(1+u^{3})$	[3.2,15.4]$\;\times \;10^{-4}$	12

The truncated $p_{T}^{2}$ range ($p_{T}^{2}>0.5\;(\mathrm{GeV}/c{)}^{2}$) is considered in the input data. ($u\equiv p_{T}^{2}$).

**Table 5.** Results of mathematical expressions learned by SR (NeSymReS (49)) on physics data (31), presented in different intervals of the variable *z* (first row).

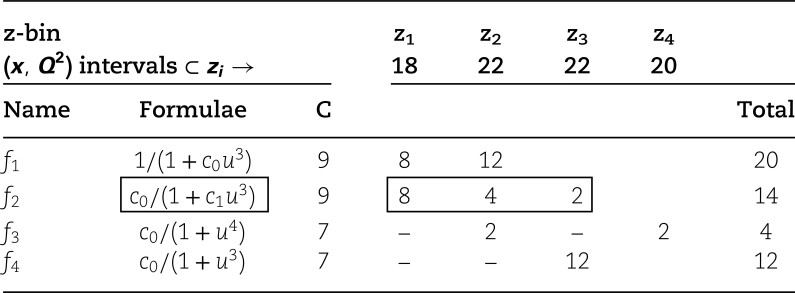

The second row presents the number of (x,Q2) intervals in each interval of *z*. The truncated $p_{T}^{2}$ range ($p_{T}^{2}>0.5\;(\mathrm{GeV}/c{)}^{2}$) is considered in the input data. The numbers in the box refer to the learned functions that have the lowest loss values, which are in the range $\left[3\times 10^{-5},3\times 10^{-3}\right]$ for $z_{1}$, $[5,25]\times 10^{-3}$ for $z_{2}$ and $[2,8]\times 10^{-5}$ for $z_{3}$. ($u\equiv p_{T}^{2}$).

**Fig. 7.**
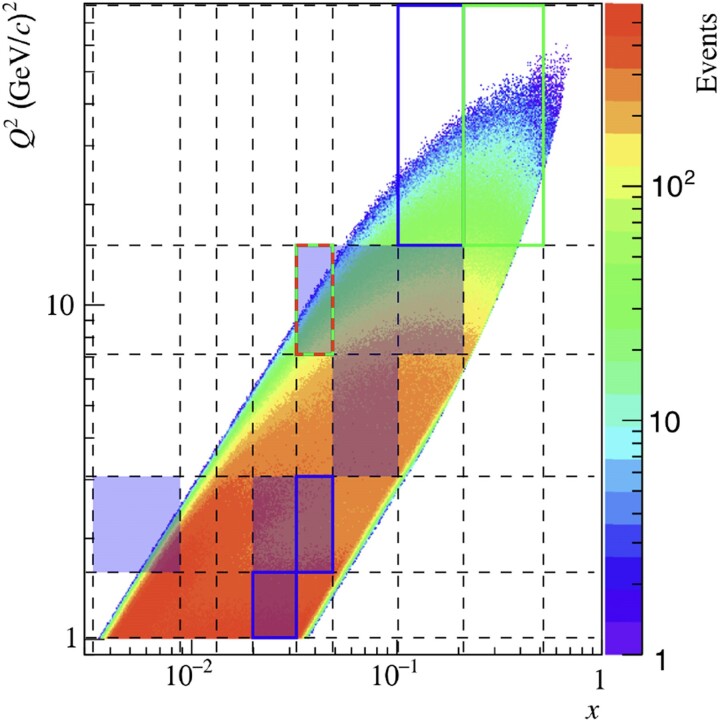
Kinematic distribution of data (scattering events) from the original analysis reported in (31) in ($x,\;Q^{2}$) intervals. The dashed lines delimit the (*x*, $Q^{2}$) intervals. The drawn boxes represent ($x,Q^{2}$) intervals where the SR model (cf. Eq. 6) is learned, for the truncated range $p_{T}^{2}>0.5$ (GeV/*c*)^2^, in the first *z* interval (0.2<z<0.3, shaded boxes), the second *z* interval (0.3<z<0.4, ($x_4,Q_1^2), ($x_5,Q_2^2$), (x_7,Q_5^2)), the third *z* interval (0.4<z<0.6, ($x_5,Q_4^2$), ($x_8,Q_5^2$)), and the fourth *z* interval (0.6<z<0.8, ($x_5,Q_4^2$)). These intervals (highlighted boxes) represent the training data sets. All other (*x*, $Q^{2}$) intervals are hence test data sets, which are used to test the validity and generalizability of the learned SR model through a fit.

**Fig. 8.**
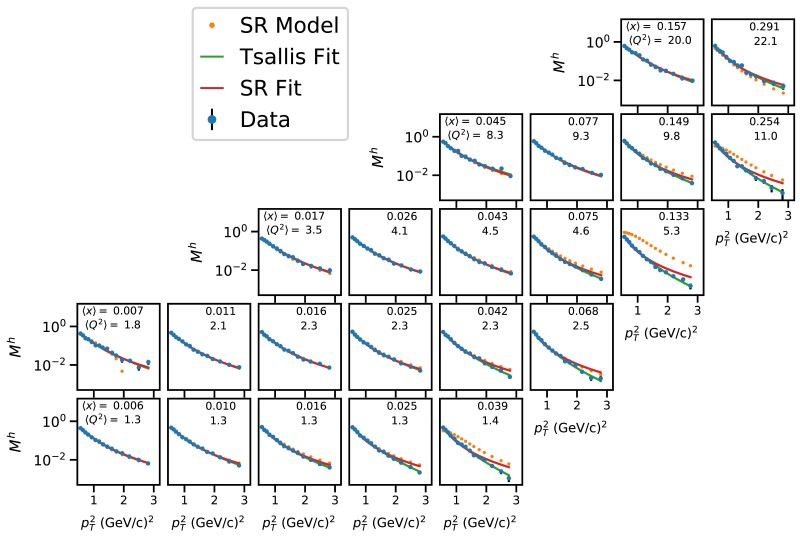
Differential multiplicities (31) of charged hadrons as a function of $p_{T}^{2}$ in ($x,Q^{2}$) intervals with $p_{T}^{2}>0.5$ (GeV/*c*)^2^ and 0.3<z<0.4. Data are compared to learned SR models and to data fits using the Tsallis model, cf. Eq. 1 and the top SR model, cf. $f_{3}(u)=c_{0}/(1+c_{1}u^{3})$ in Table 1. Statistical uncertainties are considered in the fits.

Figure 7 illustrates the kinematic phase space of the original dataset used in the analysis of (31), and highlights the $\left(x,Q^{2}\right)$ intervals where $f_{3}(u)$ (c.f., Eq. 6) was learned using SR with $p_{T}^{2}>0.5$ (GeV/*c*)^2^ in the intervals $z_{1}$ (shaded blue boxes), $z_{2}$ (blue boxes), $z_{3}$ (green boxes), and $z_{4}$ (red dashed box). These intervals represent the “training” data, whereas all others represent the “test” data, as explained in the experimental setup section. Figure 8 illustrates the results discussed in Section ‘$p_{T}^{2}>0.5\;(\mathrm{GeV}/c{)}^{2}$’. It presents the multiplicities of charged hadrons as a function of $p_{T}^{2}$, with $p_{T}^{2}>0.5$ (GeV/*c*)^2^, in ($x,Q^{2}$) intervals in the second interval of *z*, in comparison with SR models that are independently learned from individual subsets (orange markers). Also are shown fits to data using the Tsallis model (Eq. 1) and the best-learned model (Eq. 6). The Tsallis model better describes data at the largest $p_{T}^{2}$. This could be explained by the fact that the Tsallis has one additional free parameter compared to the SR model. Tables 6 and 7 report the RMSE values of the data fits using Tsallis and SR models for the full and truncated $p_{T}^{2}$ ranges, respectively. For the truncated range, the RMSE values are comparable except for the highest *x* intervals at fixed $Q^{2}$, as can be seen in Fig. 8. The RMSE values are overall higher for the full $p_{T}^{2}$ range compared to a truncated $p_{T}^{2}$ range for both models, however, the Tsallis model significantly outperfoms the SR model in all except few kinematic bins. This could be explained by the fact that the Tsallis has an additional parameter compared to the SR model. In conclusion, the Tsallis model outperforms the SR model, in particular for the full $p_{T}$ range.

**Table 6.** RMSE values of the data fits using the Tsallis (top row within $Q_{i}^{2}$) and SR (bottom row within $Q_{i^{2}}$) models by considering the full $p_{T}^{2}$ range for two intervals of *z*.

	$x_{1}$	$x_{2}$	$x_{3}$	$x_{4}$	$x_{5}$	$x_{6}$	$x_{7}$	$x_{8}$
(a) Second interval z∈[0.3,0.4]								
$Q_{5}^{2}$							0.135	1.166
							0.208	0.260
$Q_{4}^{2}$					0.055	0.058	0.036	1.329
					0.249	0.229	0.259	0.232
$Q_{3}^{2}$			0.045	0.062	0.052	0.042	1.223	
			0.273	0.237	0.201	0.204	0.157	
$Q_{2}^{2}$	0.072	0.047	0.068	0.072	0.988	1.074		
	0.326	0.253	0.244	0.180	0.148	0.118		
$Q_{1}^{2}$	0.050	0.076	0.058	1.138	1.226			
	0.267	0.259	0.217	0.203	0.180			
(b) Third interval z∈[0.4,0.6]								
$Q_{5}^{2}$							0.039	0.041
							0.089	0.131
$Q_{4}^{2}$					0.035	0.019	0.019	0.034
					0.104	0.108	0.114	0.126
$Q_{3}^{2}$			0.028	0.017	0.015	0.015	0.021	
			0.089	0.097	0.112	0.119	0.122	
$Q_{2}^{2}$	0.036	0.025	0.021	0.028	0.021	0.018		
	0.148	0.105	0.101	0.111	0.111	0.123		
$Q_{1}^{2}$	0.021	0.021	0.032	0.031	0.033			
	0.123	0.109	0.112	0.114	0.105			

The rows represent $Q^{2}$ intervals, and the columns represent *x* intervals.

**Table 7.** RMSE values of the data fits using the Tsallis (top row within $Q_{i}^{2}$) and SR (bottom row within $Q_{i}^{2}$) models by considering the truncated range $p_{T}^{2}>0.5$ (GeV/*c*)^2^ for two intervals of *z*.

	$x_{1}$	$x_{2}$	$x_{3}$	$x_{4}$	$x_{5}$	$x_{6}$	$x_{7}$	$x_{8}$
(a) Second interval z∈[0.3,0.4]								
$Q_{5}^{2}$							1.44	1.58
							1.59	1.79
$Q_{4}^{2}$					1.42	0.73	0.68	0.57
					1.84	1.04	1.01	1.40
$Q_{3}^{2}$			0.75	0.49	0.42	0.60	0.46	
			0.31	0.55	0.29	1.10	1.72	
$Q_{2}^{2}$	1.03	0.47	0.62	0.69	0.67	0.50		
	1.24	0.58	0.89	0.80	1.25	1.47		
$Q_{1}^{2}$	0.32	0.46	0.64	0.64	0.58			
	0.56	0.39	0.78	1.21	1.57			
(b) Third interval z∈[0.4,0.6]								
$Q_{5}^{2}$							1.16	0.82
							1.32	0.96
$Q_{4}^{2}$					0.35	0.49	0.19	0.62
					0.54	0.36	0.38	1.38
$Q_{3}^{2}$			0.53	0.44	0.21	0.30	0.21	
			0.54	0.31	0.28	0.50	0.86	
$Q_{2}^{2}$	0.89	0.18	0.21	0.22	0.20	0.22		
	0.91	0.41	0.22	0.23	0.56	0.93		
$Q_{1}^{2}$	0.22	0.23	0.27	0.14	0.16			
	0.30	0.10	0.39	0.39	0.95			

The rows represent $Q^{2}$ intervals, and the columns represent *x* intervals. RMSE Values are multiplied by $10^{2}$ for a better visualization.

#### $p_{T}^{2}>1\;(\mathrm{GeV}/c{)}^{2}$

The top performing expressions are summarized in Table 8. The first four functions describe at best datasets. It is noteworthy that the first expression ($f_{1}$) aligns with expectations, given the power-law behavior observed at high $p_{T}$ data, and the third expression ($f_{3}$) was already learned in the previous cases (cf. Tables 1 and 3). Finally, the last function ($f_{5}$) is the most frequently learned (in 32 out of 81 kinematic bins); nevertheless, it does not correctly describe the shape of the hadron distributions. This can be explained by the absence of constants to be optimized. However, this expression is fully compatible with previous findings since the addition of a constant to the expression $f_{5}$ recovers the basic structure in Eq. 3.

**Table 8.** Results of mathematical expressions learned by SR (NeSymReS (49)) on physics data (31) with a truncated $p_{T}^{2}$ range ($p_{T}^{2}>1\;(\mathrm{GeV}/c{)}^{2}$). ($u\equiv p_{T}^{2}$).

Name	Expression	NOF
$f_{1}(u)$	$c_{0}u^{c_{1}}$	8
$f_{2}(u)$	$1/(1+c_{0}u^{3})$	1
$f_{3}(u)$	$c_{0}/(1+c_{1}u^{3})$	1
$f_{4}(u)$	$c_{0}u^{c_{2}}/(1+c_{1}u{)}^{c_{2}}$	2
$f_{5}(u)$	$(1+u^{n}{)}^{-1},n=3,4$	32

### LHC datasets

Transverse-momentum-dependent distributions of charged hadrons measured in proton-proton collisions by the ALICE Collaboration (11–13) are evaluated using the same SR method, NeSymReS. The functions that best describe data are obtained using data in (12, 13) and are:


(7) $$\begin{array}{rl} \;f_{1}(p_{T}) & =x^{c_{0}}(1+p_{T}^{3}{)}^{-c_{0}}\\ f_{2}(p_{T}) & =c_{0}x^{c_{1}}(1+c_{2}p_{T}^{3}{)}^{-c_{1}} \end{array}$$

The two functions in Eq. 7 look different, however, $f_{2}$ can be seen as a generalized version of $f_{1}$, and fulfills the requirement of dimensional analysis which is not the case for $f_{1}$. Note that $f_{2}(p_{T})$ can be regarded as a modified version of the Hagedorn function (Eq. 2), if expressed as:


(8) $$F(p_{T})=c_{0}p_{T}^{c_{1}}{\left(1+{\left(\frac{\;p_{T}}{p_{0}}\right)}^{3}\right)}^{-c_{2}}$$

## Discussion

The frequently learned function associated with the lowest loss values that describe SIDIS data (31), in spanning different ranges of $p_{T}^{2}$ by decomposing the full $p_{T}^{2}$ range is found to be:


(9) $$f(u)=c_{0}{\left(1+c_{1}u^{3}\right)}^{-1}$$

This finding holds particular significance where a (simple and efficient) functional form is directly learned from experimentally measured transverse-momentum-dependent distributions of charged hadrons. The learned function (i) provides a fair description of the data in the kinematic phase space covered in the measurement, (ii) features simplicity in terms of the number of free parameters, aligning with the well-known simplicity of physical laws, (iii) and can be regarded as a generalization of the basic structure $(1+x^{n}{)}^{-1}$, a pattern recurrently identified in the vast majority of SR instances in this study. Although this function (Eq. 9) is learned in a limited number of intervals, it demonstrates the validity of its generalization to all kinematic intervals. Moreover, the learned function bears a striking resemblance to the Tsallis function (Eq. 1); therefore, $c_{0}$ serves as the normalization constant analogous to *A*, $c_{1}$ resembles the Tsallis parameter *T* and could be interpreted as a temperature parameter. The key distinction lies in the placement of the exponent parameter, which is associated with the variable itself rather than the sum.

The Tsallis distribution has an extra parameter *q*, which essentially controls the transition from exponential to power-law behavior. It has been observed from comparisons to data taken at RHIC and LHC that the parameter n=1/(q−1) depends on the colliding systems (e.g. d + Au, Cu + Cu, p+Pb, etc.), beam energy, particles species (51) and on the multiplicity (52) as well. This leads to the question of whether *q* could be considered a fundamental constant, as discussed in the recent perspective (53) on Tsallis statistics. The finding of this study aligns with this question, given that an equivalent parameter is not learned by SR. It is essential to acknowledge that statistical uncertainties associated with the measured data points are considered in data fits performed here. Notably, there is still no broad consensus in the research community on estimating uncertainties from ML methods, especially in deep-learning (DL)-based methods on which the SR approach is based.

The “T” parameter in the Tsallis distribution could be interpreted as the average transverse momentum $\left\langle p_{T}^{2}\right\rangle$ of the hadron distributions (31). Assuming Gaussian distributions of transverse-momentum-dependent parton distribution functions $f_{q}(x,Q^{2},k_{⊥})$ and fragmentation functions $D_{q}^{h}(z,Q^{2},p_{⊥})$ with respect to $k_{⊥}$ and $p_{⊥}$ respectively, leads to the linear relation, $\left\langle p_{T}^{2}\rangle =\langle p_{⊥}^{2}\rangle +z^{2}\langle k_{⊥}^{2}\right\rangle$, known as the Gaussian ansatz, where $k_{⊥}$ is the intrinsic transverse momentum of the scattered quark and $p_{⊥}$ is the transverse projection of the hadron’s momentum ${\vec{\;p}}_{h}$ with respect to the direction of the scattered quark. Previous measurements of hadron transverse momentum distributions (30) showed that $\left\langle p_{T}^{2}\right\rangle$ increases with increasing $Q^{2}$ (at fixed values of *x*). An effect that is more visible at high $z^{2}$ where the contribution of the quark intrinsic transverse momentum to hadron transverse momentum is enhanced. The dependence of the parameter ($1/c_{1}$) obtained from data fits using the learned function by SR, cf. Eq. 6, on $z^{2}$ is found to validate the linear relation of the Gaussian ansatz and increases with higher $Q^{2}$, and thus can be interpreted as the average transverse momentum of hadron distribution.

Despite the remarkably fast developments in symbolic regression, it remains at an early stage and requires further tests and applications to be established as a scientific discovery tool; it has been questioned in (47) how SR methods work and for which datasets they fail. For example, it is intriguing how a simple polynomial equation, which is trivial for linear methods, fails to be learned by very intricate complex SR approaches. In addition, the application of SR to experimental data is very limited. To the best of our knowledge, this study represents the first application of an ML-based method, namely SR, to hadron production study in high-energy physics and to such a multi-dimensional dataset that is experimentally measured. Learning a mathematical function from inherently noisy experimental data that closely resembles the Tsallis statistical distribution demonstrates that SR can deliver convincing results, which is a major achievement towards establishing it as a discovery tool, holds promise to extend its applicability to other observables in high-energy physics and beyond, and finally advocates SR as one of the most potential candidate for advancing sciences in the AI era.

## Conclusions

This study investigates the application of symbolic regression to an actual physics problem that is currently under investigation through experiments and theoretical studies. With the core objective of extracting a mathematical expression from experimental data and evaluating its alignment with established formulas, the finding presents promising results. Symbolic regression, as a facet of interpretable machine learning, demonstrates its potential to discover analytical models directly from data. Moreover, these results underscore the pivotal role of interpretable machine learning in aiding theorists in comprehending intricate phenomena.

## Data Availability

The data supporting this study’s findings were measured by the COMPASS Collaboration (31). They are openly available in the Durham High Energy Physics Database (HEPData) [https://www.hepdata.net/record/ins1624692]. The Software used in this study is presented in (49) and is openly available.

## Notes

a PYTHIA and HERWIG are physics event generators developed and used in high energy physics.

b

cos(αx) is equivalent to cos(αx+1−1) or cos(αx)*1 or (2−1)*cos(αx), etc.

c The equations in training examples are succinct and do not include any additional terms that cancel-out.

d NLP stands for Natural Language Processing.

e The prefix form, also known as prefix notation or Polish notation (45), is a mathematical notation in which an operator precedes its operands, e=∘*sin(x)+∘*x≡{+,*,∘,sin,x,*,∘,x}.

f A subset is a one-dimensional $p_{T}^{2}$-dependent hadron distribution measured in a defined ($x_{i_{x}}$, $Q_{i_{q}}^{2}$, $z_{i_{z}}$) interval unless otherwise specified, where $i_{x}\in [1,8],i_{q}\in [1,5],i_{z}\in [1,4]$.
